# Antimelanogenic Effect of an *Oroxylum indicum* Seed Extract by Suppression of MITF Expression through Activation of MAPK Signaling Protein

**DOI:** 10.3390/ijms19030760

**Published:** 2018-03-07

**Authors:** Peijun Zhao, Md Badrul Alam, Hongyan An, Hee-Jeong Choi, Yeong Ho Cha, Chi-Yeol Yoo, Hyo-Hyun Kim, Sang-Han Lee

**Affiliations:** 1 Department of Food Science and Biotechnology, Graduate School, Kyungpook National University, Daegu 41566, Korea; laputaily@hotmail.com (P.Z.); anhongyan@outlook.com (H.A.); choi930302@gmail.com (H.-J.C.); yeongho9205@naver.com (Y.H.C.); yousee0581@naver.com (C.-Y.Y.); 2 Food and Bio-Industry Research Institute, Kyungpook National University, Daegu 41566, Korea; mbalam@knu.ac.kr; 3 MR Innovation Co., Ltd., KNU Technopark, Daegu 41566, Korea; kimhyohyun35@naver.com

**Keywords:** antimelanogenic, *Oroxylum indicum*, mitogen-activated protein kinase (MAPK), microphthalmia-associated transcription factor (MITF)

## Abstract

In this study, the antimelanogenic effect of an ethyl acetate fraction of *Oroxylum indicum* Vent. seeds (OISEA) and its underlying mechanisms in melan-a cells were investigated. Antimelanogenesis activity was confirmed by assessing inhibition of tyrosinase activity and melanin content in the cells. Both transcriptional and translational expression of microphthalmia-associated transcription factor (MITF), tyrosinase, and tyrosinase related protein-1 and 2 (TYRP-1 and TYRP-2), were also examined. The results depicted that pretreatment of OISEA significantly inhibits not only tyrosinase activity, but melanin production and intracellular tyrosinase activity. By repressing the expression of tyrosinase, TYRP-1, TYRP-2, and MITF, OISEA interrupted melanin production. Additionally, OISEA interfered with the phosphorylation of p38, extracellular signal-regulated kinase 1/2 (ERK1/2), and c-Jun N-terminal kinase (JNK), with the reversal of OISEA-induced melanogenesis inhibition after treatment with the specific inhibitors SB239063, U0126, and SP600125. Overall, these results suggest that OISEA can stimulate p38, ERK1/2, JNK phosphorylation, and subsequent suppression of melanin, leading to the inhibition of melanogenic enzymes and melanin production, possibly owing to the presence of polyphenolic compounds.

## 1. Introduction

Melanin, a multifunctional biopolymer, is synthesized in melanocytes and transferred to keratinocytes [1]. It has many fundamental physiological functions, including contribution to the color of the skin and protection of the skin from ultraviolet radiation (UVR)-induced damage. However, abnormal hyperpigmentation in human skin, such as melasma, freckles, and chloasma, is a serious aesthetic problem [2]. Hence, the inhibitors of melanogenesis have gained substantial attention in clinical and cosmetic research. Currently, several cutaneous depigmentation agents, such as kojic acid, arbutin, and ellagic acid, isolated from natural resources, are used as cosmetic agents [3].

Tyrosinase (copper-containing enzyme) plays a pivotal role in melanogenesis. It catalyzes the two rate-limiting steps of melanin biosynthesis: first, production of 3,4-dihydroxyphenylalanine (DOPA) by hydroxylation of tyrosine, referred to as monophenolase activity, and second, formation of dopaquinone from oxidation of DOPA, which is known as diphenolase activity [4]. The inhibition of tyrosinase function is an efficient approach in the treatment of hyperpigmentary disorders. Melanogenesis is regulated by numerous physiological and pathological effectors, such as ultraviolet radiation, melanocortin, NO, sex steroids, and various growth factors [5]. Evidence indicates that the UV-induced generation of reactive oxygen species (ROS) and reactive nitrogen species (RNS) in melanocytes, and induction of melanogenesis, are perhaps through the upregulation of tyrosinase [6]. Melanogenesis is also modulated by various signal transduction pathways, such as the cyclic adenosine monophosphate (cAMP)/protein kinase A (PKA) pathway, which is viewed as being among the most important regulators of melanogenesis [7]. Furthermore, mitogen-activated protein kinase (MAPK) signaling through extracellular signal-regulated kinases (ERK), c-Jun N-terminal kinase (JNK), and p38 MAPK leads to microphthalmia-associated transcription factor (MITF) ubiquitination and degradation, and plays a crucial role in lessening melanin production [8].

**Figure 1:**
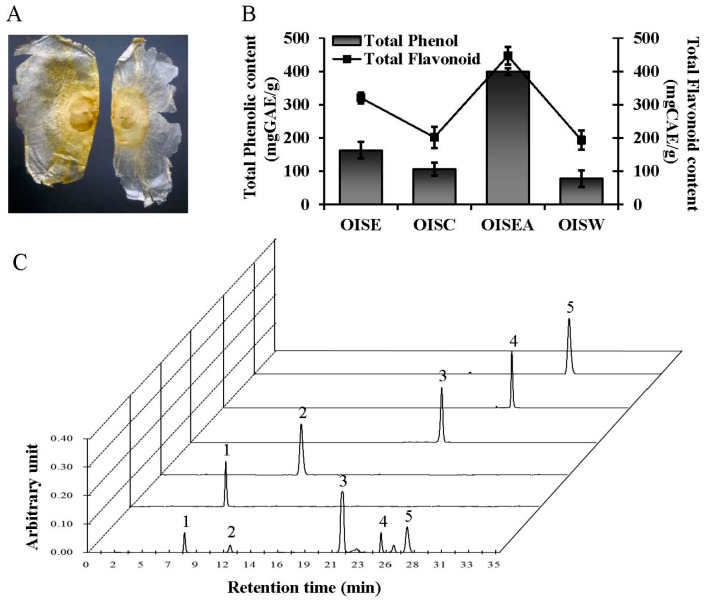
Characteristics of *Oroxylum indicum* seed. (**A**) A classical feature of *Oroxylum indicum* seed. (**B**) Total phenolic and flavonoid contents are shown. (**C**) A representative high-performance liquid chromatography (HPLC) profile of *Oroxylum indicum* seed extract with standards monitored at 280 nm. Gallic acid (Peak 1), caffeic acid (Peak 2), baicalein (Peak 3), chrysin (Peak 4), and oroxylin B (Peak 5) are also presented. OISE: ethanol extract; OISC: chloroform fraction; OISEA: ethyl acetate fraction; and OISW: aqueous fractions of *Oroxylum indicum* seed.

Being safe and mostly free of side effects, natural products are considered suitable for the exploitation of effective and prophylactic skin depigmentation agents in cosmetic research and development. To exploit novel ingredients displaying tyrosinase-inhibitory activities as leading structures for inhibitors of melanin biosynthesis, many extracts/fractions of medicinal plants have been assessed using a tyrosinase inhibition assay. Among these plants and their extracts, the ethanol extract of the seeds of *Oroxylum indicum* Vent. (OIS) (Figure 1A) exhibited potent antioxidant and tyrosinase-inhibiting activities. *O. indicum*. (Bignoniaceae) is a tree commonly found in tropical countries, such as China, India, Japan, Sri Lanka, and Malaysia. Different parts of this plant are rich in polyphenolics, such as chrysin, apegenin, prunetin, baicalein and its glucosides, sitosterol, oroxindin, biochanin-A, ellagic acid, scutellarein, tetuin, antraquinone, and aloe-emodin [9,10,11,12]. In Ayurveda and folk medicine, various parts of the plant are practiced for the treatment of different ailments, such as cancer, diarrhea, fever, ulcer, and jaundice [13]. Recent studies disclosed that the plants had anti-inflammatory, antiulcer [14], antiproliferative, anticancer [10], antioxidant [12], and immunostimulant [15] properties. However, there is no scientific foundation for *O. indicum*-based folk cosmetics. In the present study, we investigated the inhibitory effect of an OIS extract on tyrosinase and melanogenesis, as well as its underlying molecular mechanism, in melan-a cells.

## 2. Results

### 2.1. High-Performance Liquid Chromatograms of the Ethyl Acetate Fraction of Oroxylum indicum Vent. Seeds (OISEA)

Recent research suggested that polyphenolics from the natural sources are viewed as the most promising therapeutic agents for the prevention of various skin disorders, such as hyperpigmentation, aging, and cancer. Various polyphenols, exhibiting antioxidant potential, are playing an important role in controlling skin balance that can be utilized as superlative natural compounds for a variety of skin troubles and whitening. Therefore, it is worthwhile to resolve the total phenolic and flavonoid content in the plant taken for the study (Figure 1B). In this study, total phenolic and flavonoids contents of OISEA were 399.92 ± 132.78 mg gallic acid equivalent (GAE)/g and 446.77 ± 116.87 mg caffeic acid equivalent (CAE)/g, respectively. Interestingly, the HPLC chromatogram (Figure 1C) showed that the OISEA extract possessed various polyphenolics compared with the same retention times as the following standard polyphenolics: gallic acid (8.17 min), caffeic acid (12.25 min), baicalein (22.15 min), chrysin (25.44 min), and oroxylin B (27.53 min). Using the peak areas of standards with known concentrations, the amounts of these polyphenolic compounds in OISEA were calculated. As shown in Figure 1C, the contents of gallic acid, caffeic acid, baicalein, chrysin, and oroxylin B were 2.565%, 1.059%, 22.654%, 2.445%, and 3.136%, respectively.

### 2.2. Inhibitory Effects of OISEA on Tyrosinase Activity

**Figure 2:**
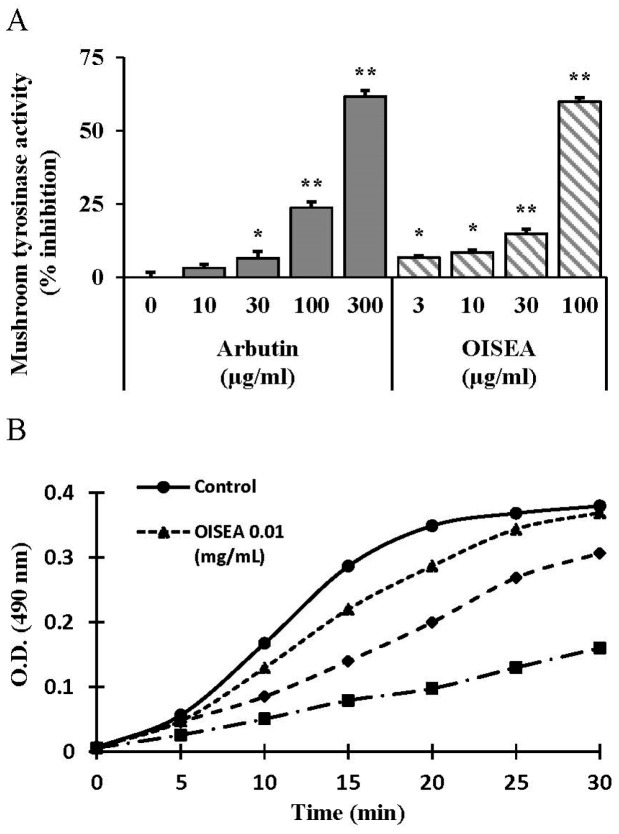
Inhibitory effects of OISEA on mushroom tyrosinase activity. (**A**) Different concentrations of OISEA or arbutin were incubated with the same units of mushroom tyrosinase. Following incubation, amounts of dopachrome produced were determined at 490 nm spectrophotometrically. (**B**) Effects of OISEA on the monophenolase activity of tyrosinase. Enzyme activity was tested in the presence of l-tyrosine, as substrate. Results are presented as the means ± SD of three experiments. * *p* < 0.05, ** *p* < 0.01, versus non-treated controls, Arb: Arbutin.

In our ongoing research to identify melanogenesis inhibitors from natural sources, the ethanol extract, along with its various organic fractions of OIS, were found to hinder mushroom tyrosinase activity (Figure 2A and Figure S1). To the best of our knowledge, as no kinetic study on this inhibitory effect has been conducted earlier, to evaluate the tyrosinase inhibitory effect of the ethyl acetate fraction of OIS (OISEA), we tested its effect on the monophenolase activity of the enzyme by fixing the kinetic parameters (Figure 2B). When tyrosinase initiated the enzymatic reaction through the action on l-tyrosine, a distinguished lag period, as a characteristic of monophenolase activity, was observed (Figure 2B).

### 2.3. Effects of the OISEA on Melanogenesis

**Figure 3:**
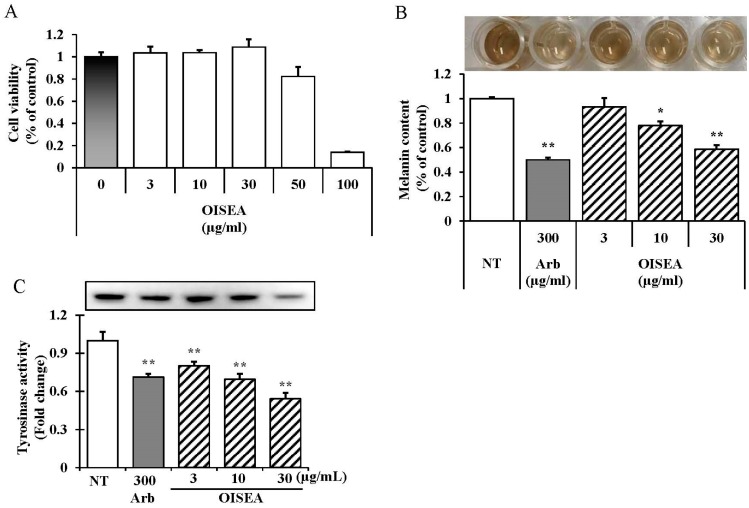
Effects of OISEA on melanogenesis in melan-a cells. Cells were cultured with OISEA (3–10 μg/mL) for 3 days. (**A**) Cytotoxicities, (**B**) melanin contents, and (**C**) tyrosinase activities were measured as described in Materials and Methods. Experiments were performed in triplicate, and results are presented as means ± SD. * *p* < 0.05, ** *p* < 0.01, NT: No treatment; Arb: Arbutin.

Melanin levels in melan-a cells were essayed after exposure to OISEA at concentrations of 3–30 µg/mL, which did not exhibit any cytotoxic effect (Figure 3A). We observed that melanin levels were significantly remittent after exposure to OISEA in a dose-dependent manner (Figure 3B, 3–5 columns). Furthermore, to examine the inhibitory mechanisms of OISEA on melanogenesis more precisely, l-DOPA zymography was performed to accomplish the intracellular tyrosinase activity in melan-a cells. As shown in Figure 3C (3–5 columns), treatment with OISEA at indicated concentrations increased tyrosinase inhibition. These results suggest that OISEA inhibits melanogenesis in melan-a cells.

### 2.4. Effects of OISEA on the mRNA and Protein Expression of Melanogeneis-Related Genes

**Figure 4:**
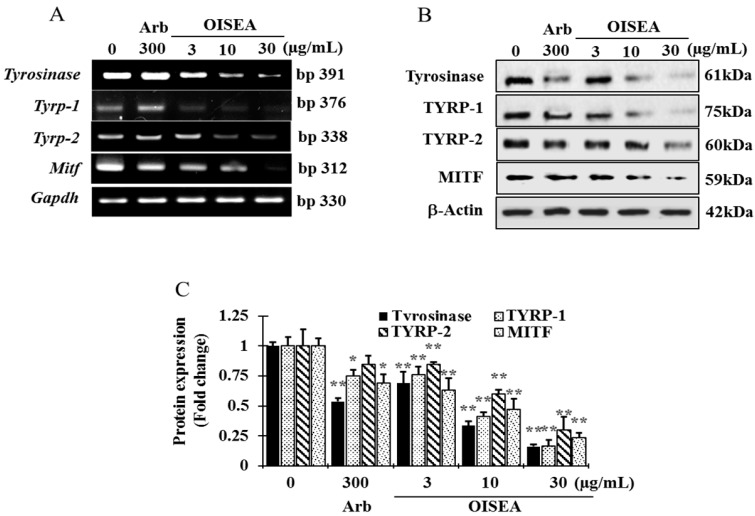
Effect of OISEA on the levels of melanogenesis related mRNA and proteins in melan-a cells. (**A**) Cells (5 × 10^5^ cells/mL) were cultured for 24 h; the medium was replaced with fresh medium containing the indicated concentration of OISEA or arbutin for 24 h, and the mRNA was extracted using TRIzol. mRNA expression was analyzed by reverse transcription-polymerase chain reactions. (**B**) Cells (5 × 10^5^ cells/mL) were cultured for 24 h; the medium was replaced with fresh medium containing the indicated concentrations of OISEA or arbutin for 3 days. Total cell lysates were extracted and assayed by Western blotting using antibodies against tyrosinase, TYRP-1, TYRP-2, and MITF. Equal amounts of protein loading were confirmed using β-actin. (**C**) Statistical analysis of the band intensity of tyrosinase, TYRP-1, TYRP-2, and MITF obtained by Western blot analysis. Results are presented as means ± SD. * *p* < 0.05, ** *p* < 0.01, versus the non-treated group.

To elucidate the mechanics of OISEA on melanogenesis, we next examined the expression of tyrosinase, TYRP-1, TYRP-2, and MITF in melan-a cells after treatment with OISEA (3, 10, or 30 μg/mL) for 4 days. OISEA suppressed the mRNA expression of *Mitf* and its downstream genes *tyrosinase, Tyrp-1*, and *Tyrp-2* (Figure 4A). In addition, 30 μg/mL OISEA importantly shortened tyrosinase, TYRP-1, TYRP-2, and MITF protein levels in a dose-dependent manner compared with the control (Figure 4B,C). These results evoke that OISEA ameliorated melanogenesis by conquering the expression of tyrosinase, TYRP-1, and TYRP-2 through the deactivation of MITF in melan-a cells.

### 2.5. Effects of OISEA on MAPK-Dependent Signaling in Melan-a Cells

**Figure 5:**
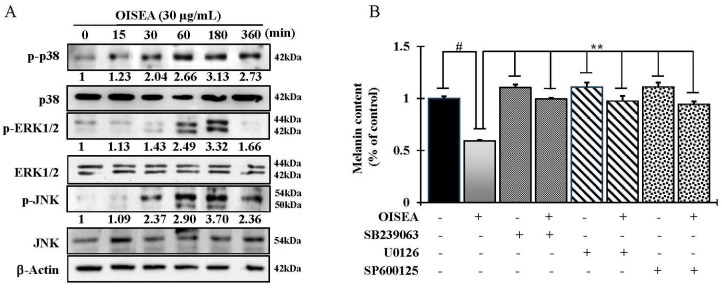
Effect of HLA on MAP kinase-dependent signaling in melan-a cells. (**A**) Cells (5 × 10^5^ cells/mL) were cultured for 24 h, and the medium was replaced with fresh medium containing various concentrations of test compounds or arbutin for the indicated times. Phosphorylation of JNK, ERK, and p38 MAPK was analyzed using phospho-specific JNK, ERK, and p38 MAPK antibodies. Equal protein loading was checked using β-actin antibodies. Numbers (each phospho-antibody) mean the relative band intensity. (**B**) OISEA was cotreated with selective inhibitors of p38 (SB239063), ERK (U0126), and JNK (SP600125) signaling molecules in melan-a cells. Melanin content was determined. Each determination was made in triplicate, and the data represent the means ± SD. ^#^ *p* < 0.05, ** *p* < 0.01, versus the control group.

Next, to identify the particular mechanisms inherent, the antimelanogenic activity of OISEA, Western blot analysis of p38, ERK, and JNK signaling proteins were studied in melan-a cells. OISEA at nontoxic concentrations efficaciously actuated the phosphorylation of p38, ERK, and JNK in a time-dependent manner (Figure 5A). Furthermore, co-treatment of OISEA with p38 inhibitor (SB239063), ERK1/2 inhibitor (U0126), and JNK inhibitor (SP600125) significantly reversed OISEA-induced decrease in melanin content (Figure 5B).

## 3. Discussion

For the purpose of applications of cosmetics and food industry of useful herbal and/or Oriental plants, it is important to carry out isolation, identification, characterization, and evaluation of functionality and mechanistic studies, in vitro and in vivo. Originally, *O. indicum* is a commonly used herbal medicine in Ayurveda. The roots, leaves, and stems of *O. indicum* have been used individually, or as components of other Ayurvedic drug preparations for the treatment of various disorders. A few scientific studies have been conducted on the possible cosmetic applications of the various parts of the plant, and hence, extensive investigations are required to exploit their therapeutic utility. In the present study, ethyl acetate fraction of *Oroxylum indicum* Vent. seeds (OISEA) was observed to cause significant inhibition of tyrosinase activity. Interestingly, OISEA acted as a tyrosinase inhibitor at its monophenolase phase. The lag period was dependent on enzyme and substrate concentrations in the reaction medium, and was minimized, or even abolished, owing to the presence of the catalytic amount of transition metal ions or *o*-diphenols [16]. However, the lag phase is known to be extended by some monophenolase inhibitors, such as glabrene and *p*-alkoxybenzoic acid. OISEA extended the lag phase by 20 min compared with the control, particularly when its concentration was 100 μg/mL. Our results lead us to hypothesize that OISEA were attached to a site different from the active site, and hindered the binding of substrate to the enzyme through steric hindrance or by changing the protein conformation [17].

Tyrosinase plays a central role in the melanin biosynthesis, as it converts l-tyrosinase to l-DOPA, which is then oxidized to from dopachrome. Hence, mushroom tyrosinase inhibition assay is a widely acceptable screening method to develop the potential inhibitors of melanogenesis [5]. Here, OISEA showed substantial inhibitory effects on mushroom tyrosinase activity (Figure 2A). Furthermore, OISEA treatment significantly attenuated the intracellular tyrosinase activity and eventually suppressed the melanin production in melan-a cells (Figure 2B,C). Tyrosinase inhibitory activities of OISEA are involved in its phenolic acids and antioxidant activities, which are close to that of an earlier study [18], where it was described that TYR inhibitory activity is related to the hydroxyl group of phenolic compounds. This group forms hydrogen bonds with enzyme active sites to enhance steric hindrance, conformation changes, and directly take enzymatic activity. Recent studies reported that gallic acid and caffeic acid are effective TYR inhibitors [2,19]. A potent suppressive impact on tyrosinase was also shown by a leaf extract from *Morus alba*, commonly used in traditional medicine, mainly due to the presence of phenolic compounds in the extract [20].

Noteworthy that MITF and its downregulation proteins, such as tyrosinase, TYRP-1, TYRP-2, MITF, ASIP (agouti signaling protein), and MGRN1 (mahogunin ring finger-1) [21], are viewed as the regulators of melanogenesis, and tyrosinase inhibitors may control melanin production by suppressing their mRNAs and protein levels in cells. In the present study, OISEA treatment significantly suppressed the mRNA level of MITF, and its downstream targets, such as tyrosinase, TYRP-1, and TYRP-2 (Figure 3), as well as their proteins levels. The current results indicate that OISEA deteriorated melanogenesis by controlling the expression of tyrosinase, TYRP-1, and TYRP-2 via a deactivation pathway of MITF in melan-a cells. A recent study revealed that the extract from the small shrub *Arthrophytum scoparium* presented tyrosinase and tyrosinase-regulated gene repression, predominantly due to the downregulation of microphthalmia-associated transcription factor (Mitf) and melanocortin 1 receptor (Mc1R) [22]. The MAP kinase family, including extracellular signal-regulated kinase (ERK)1/2, c-Jun N-terminal kinase (JNK), and p38 MAPK, plays a crucial role in melanogenesis. Investigations imply that p38 MAPK is an intracellular signaling molecule vital to pigment formation [23], and the ERK1/2 and JNK are also related to the downregulation of melanogenic processes [8]. We therefore analyzed the influence of OISEA treatment on the activation of ERK, JNK, and p38 MAPK, to further investigate the molecular mechanisms engaged in the melanogenesis in melan-a cells, by Western blot assay. In this study, OISEA treatment significantly enhanced the phosphorylation of p38, ERK, and JNK in a time-dependent manner. This finding proposed that OISEA-induced antimelanogenesis in melan-a cells through MAPKs mediated pathways. Furthermore, co-treatment with OISEA and specific inhibitors (SB239063 for p38, U0126 for ERK1/2, and SP600125 for JNK) significantly reversed OISEA-induced melanin content (Figure 4B).

Antioxidants are also well acknowledged to play a pivotal role in the antimelanogenesis [21]. In this study, OISEA had significant scavenging capacity of DPPH^•^ (a stable organic nitrogen radical) and ABTS^•+^ (a mixed electron and hydrogen atom transfer assay) in a dose-dependent manner (Figure S2). In addition, OISEA was found to have a strong electron donating capacity to reduce Cu and Fe metal, in cupric reducing antioxidant capacity (CUPRAC) and ferric reducing antioxidant power (FRAP) assay, respectively, and acted in a concentration-dependent fashion (Figure S3). This can be compared by Pearson’s correlation analysis between antioxidant and antimelanogenic potentials. It is noteworthy that OISEA is rich in polyphenolic compounds [9,10]. Recent studies have shown that polyphenolics with antioxidative potential showed repressive effects on melanogenesis in B16 cells [21,24]. In this study, HPLC analysis revealed that gallic acid, caffeic acid, baicalein, chrysin, and oroxylin A were present in OISEA. Interestingly, gallic acid, caffeic acid, baicalein, and chrysin have been accounted to have both antioxidative and antimelanogenic effects [19,25,26,27], whereas oroxylin B had no such reports. A literature review revealed that baichalein and chrysin inhibited melanin production 20% and 60%, at 50 μM and 100 μM, respectively. In the present study, OISEA suppressed melanin production by 58% at 30 μg/mL, suggesting that the cluster of polyphenolics in OISEA was providing the synergistic effects.

## 4. Materials and Methods

### 4.1. Plant Materials and Extraction

OIS (Figure 1A) were bought from a Chinese traditional herb store in Zhengzhou, China. The plant material was taxonomically identified, and the voucher specimen (#2016-Oi) was retained in our laboratory for future reference. The air-dried OIS (30 g) were pulverized and extracted with 100% ethanol for 24 h three times in a shaking incubator at 45 °C. Next, the supernatant was decanted through a filter paper (No. 1 Whatman Schleicher Schuell, Keene, NH, USA) and lyophilized with a freeze dryer (Ilsinbiobase, Goyang, Korea). The residue considered as ethanol extract (OISE). Approximately twenty grams of OISE was suspended in 200 mL of deionized H_2_O, followed by consecutive partitioning with chloroform and ethyl acetate using separatory funnels, in a stepwise manner. Then, vacuum filtration and concentration using a rotary vacuum evaporator (Tokyo Rikakikai Co. Ltd., Tokyo, Japan) were carried out, producing a chloroform fraction (OISC) (2.98 g), ethyl acetate fraction (OISEA) (4.52 g), and aqueous fraction (4.21 g). Finally, all extract/fractions had subjected to dissolution in deionized H_2_O at a concentration of 30 mg/mL.

### 4.2. Drugs and Chemicals

Arbutin, sodium hydroxide, *O*-tetradecanoyl phorbol-13-acetate (TPA), thiazolyl blue tetrazolium bromide (MTT), Tween-20, and l-tyrosine were purchased from Sigma-Aldrich Co. (St. Louis, MO, USA). Other chemicals were of special grade, and were commercially available.

### 4.3. Chemical Compound Analysis by High-Performance Liquid Chromatography

An Agilent 1200 chromatographic system equipped with a quaternary pump, an UV–vis diode-array detector, an automatic injector, and ChemStation software (Palo Alto, CA, USA) was operated for the high-performance liquid chromatography (HPLC) separation, identification, and quantification of polyphenol compounds. A 0.45-µm nylon filter (E0034, Análisis Vínicos, Tomelloso, Spain) was used to filter the sample before analysis. All analyses were performed in triplicate. The quantification of phytoconstituents in ethanol extracts was performed by the HPLC method on a base deactivated RP [28]. Complete separation of the phytoconstituents was achieved using a Zorbax C18 column (150 × 4.6 mm, 5 µm particle size; Agilent Technologies, Santa Clara, CA, USA) maintained at 30 °C. The mobile phase consisted of water/methanol/acetonitrile/ortho phosphoric acid (60:30:38:1, *v*/*v*/*v*/*v*). The flow rate was 1 mL/min, and the injection volume was 10 µL. The wavelengths of detection were 262 nm. The isocratic method was run for 35 min, and compounds were distinguished by comparing their retention time with those of the available pure standards. The external calibration method was used for quantification by comparing their areas with those of standards of gallic acid, caffeic acid, baicalein, chrysin, and oroxylin B.

### 4.4. Tyrosinase Inhibition Assay

The mushroom tyrosinase inhibitory activity of OISEA was determined by spectrophotometry as described previously, with minor modifications [29]. The reaction mixture containing 100 μL of 0.1 M phosphate buffer (pH 6.5) with or without sample, 50 μL of 1 mM l-tyrosine, and 50 μL of mushroom tyrosinase (200 units/mL) was added into a 96-well microplate (SPL, Pocheon, Korea). The linear increase in OD_490_ was measured at room temperature.

### 4.5. Cell Culture and Cell Viability Assay

Melan-a was obtained from Dorothy C. Bennett (St George’s, University of London, London, UK) and was cultured at 37 °C under 5% CO_2_ in RPMI 1640 supplemented with 10% fetal bovine serum (FBS, Hyclone, Utah, UT, USA), streptomycin–penicillin (100 µg/mL each), and 200 nM TPA (a potent tumor promoter) every 3 days, up to a maximum of 40 passages. The cytotoxicity of OISEA was measured by using the MTT assay based on the reduction of MTT to formazan. In brief, cells were seeded in 96-well plates (1 × 10^5^ cells/well), incubated overnight, and exposed to various concentrations of OISEA for the next 24 h, followed by the addition of 10% MTT solution to each well, and incubated at 37 °C for 1 h. After removing the media, the plate was washed twice with phosphate-buffered saline (pH 7.4). Dimethyl sulfoxide (DMSO) was used to solubilize the formazan and measured at 570 nm by a plate reader (Victor3, Perkin Elmer, Waltham, MA, USA), and the cell viability was analyzed as a percentage.

### 4.6. Cellular Melanin Content in Melan-A Cells

Cells (1 × 10^5^ cells/mL) were plated into a 24-well plate (BD Falcon, Bedford, MA, USA), and permitted to attach overnight. The old media was removed by suction, followed by the addition of fresh media containing predetermined concentrations of OISEA, and culture for 72 h. Thereafter, cells were rinsed twice with PBS, lysed in 1 N NaOH, and measured at 405 nm by a microplate reader (VICTOR3, Perkin Elmer) for melanin content. Arbutin was used as a positive control.

### 4.7. Analysis of Intracellular Tyrosinase Activity by Zymography

Tyrosinase zymography was executed as previously described [4]. Cells were pretreated with or without OISEA for 72 h. Cells were washed with PBS twice and lysed with RIPA buffer supplemented with phosphatase and protease cocktail inhibitors. A BCA protein assay kit (Pierce Biotechnology, Rockford, IL, USA) was used to measure total protein concentration. A 50 μg sample of total proteins were separated by 10% SDS-PAGE (sodium dodecyl sulfate-polyacrylamide gel electrophoresis) and the gels were incubated for 30 min using phosphate buffer (pH 6.8) with mild shaking. Finally, L-DOPA in phosphate buffer (pH 6.8) was used to stain the gel for 1 h.

### 4.8. Reverse Transcription-Polymerase Chain Reaction (RT-PCR)

Total RNA was pulled out using TRIzol (Invitrogen Co., Carlsbad, CA, USA), according to the manufacturer’s instructions. To prepare a cDNA pool from RNAs, RT-&GO Mastermix (MP Biomedicals, Seoul, Korea) protocol was adopted using 2 µg of total RNA, and the product was considered as the PCR template. RT-PCR was achieved using a PCR Thermal Cycler Dice TP600 (TAKARA Bio Inc., Otsu, Japan) using the primer sequences listed in Table 1. PCR products were separated on 2% agarose gel in Tris-Acetate-EDTA (TAE) buffer at 100 V for 30 min and visualized by ethidium bromide (Bio-Rad Laboratories, Hercules, CA, USA) staining.

### 4.9. Western Blotting Analysis

Melan-a cell lysates were mixed with sample buffer (250 mM Tris-HCl (pH 6.8), 0.5 M DTT, 10% SDS, 0.5% bromophenol blue, 50% glycerol, 5% 2-mercaptoethanol), and denatured at 100 °C for 5 min using a standard protocol. A 10% of SDS-PAGE was used to separate the sample proteins (50 µg). Following electrotransfer to nitrocellulose membranes (Whatman, Dassel, Germany), the membranes were immersed overnight in a mixture containing antibodies and 5% skim milk. Primary antibodies, such as anti-tyrosinase, anti-TRP-1, anti-TRP2, anti-MITF, p-CREB, and total CREB and β-actin, were from Bioworld Technology (St. Louis Park, MN, USA). Secondary antibodies (anti-goat IgG-horse radish peroxidase (HRP) and anti-mouse IgG-HRP) were purchased from Santa Cruz. The resulting reaction was exposed using an ECL solution system (Perkin Elmer).

### 4.10. Statistical Analysis

Results were measured using one-way ANOVA, and are denoted as means ± SD. The analysis was achieved using SPSS for Windows Ver. 10.07 (SPSS, Chicago, IL, USA), and statistical significance was set at *p* < 0.01 or <0.05.

## 5. Conclusions

**Figure 6:**
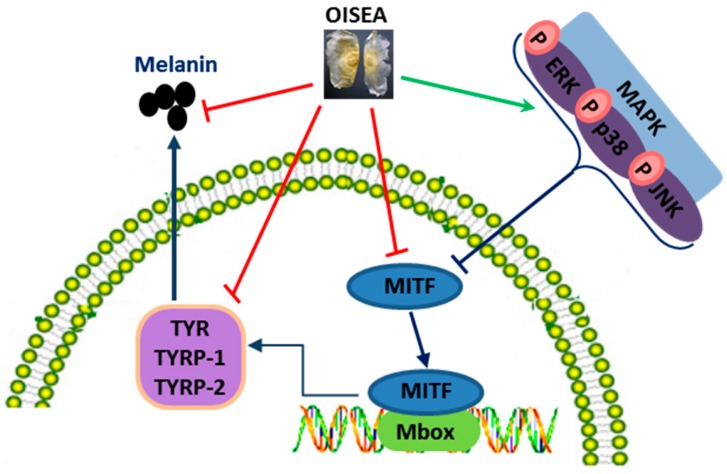
A proposed molecular mechanism of OISEA against melanogenesis process.

The present study described, for the first time, the inhibitory effects of OISEA on melanogenesis in melan-a cells (Figure 6). The noteworthy aspects of the present study are as follows. (a) OISEA was found to repress melanogenesis powerfully and inhibit mushroom tyrosinase activity. (b) The mechanisms through which OISEA extenuated melanin production by downregulating MITF expression through the interference with ERK1/2, JNK, and p38 phosphorylation, besides decreasing tyrosinase, TYRP-1, and TYRP-2 levels. (c) Furthermore, our findings suggest that the observed suppressive effect of OISEA on melanin might be due to the presence of the cluster of polyphenolics which provide the synergistic effect. Therefore, it can be practiced for the exploitation of therapeutic or novel whitening agents in the cosmetic, nutraceutical, or food industry.

## Figures and Tables

**Table 1:** List of the primer used in this study.

Gene Name		Sequences
*Tyrosinase*	Forward	CCC AGA AGC CAA TGC ACC TA
	Reverse	ATA ACA GCT CCC ACC AGT GC
*TRYP-1*	Forward	GCT GCA GGA GCC TTC TTT CT
	Reverse	AGA CGC TGC ACT GCT GGT C
*TYRP-2*	Forward	GGA TGA CCG TGA GCA ATG GC
	Reverse	CGG TTG TGA CCA ATG GGT GC
*MITF*	Forward	CAG GCT AGA GCG CAT GGA CT
	Reverse	CTC CGT TTC TTC TGC GCT CA
*GAPDH*	Forward	GC GAG ACC CCA CTA ACA TCA
	Reverse	GAG TTG GGA TAG GGC CTC TCT

## Supplementary Materials

Supplementary materials can be found at http://www.mdpi.com/1422-0067/19/3/760/s1.
