# Supplementary material for: Antimelanogenic Effect of an Oroxylum indicum Seed Extract by Suppression of MITF Expression through Activation of MAPK Signaling Protein
Source: Int J Mol Sci. 2018 Mar 7;19(3):760. doi: 10.3390/ijms19030760 (PMC5877621; doi:10.3390/ijms19030760)
Supplement: Supplementary file 1 [file ijms-19-00760-s001.pdf]

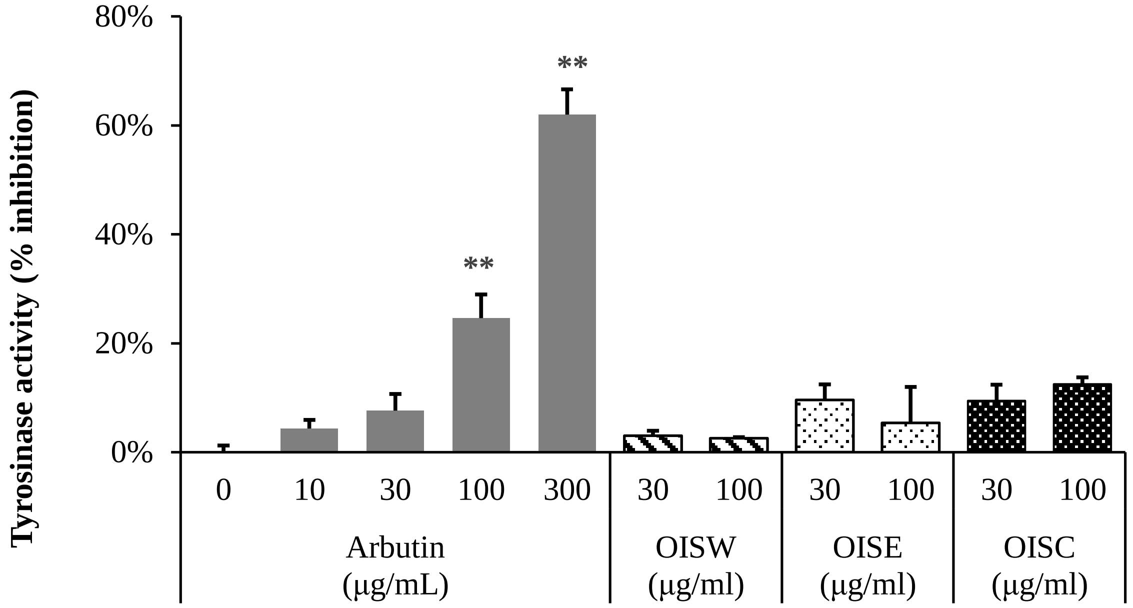

Figure S1: Mushroom tyrosinase inhibition activities of methanolic extract and various organic solution fractions of *Oroxylum indicum* seed . Values are expressed as the mean ± SD (n=3). **$p < 0.01$, significantly different from control, using student's t-test.

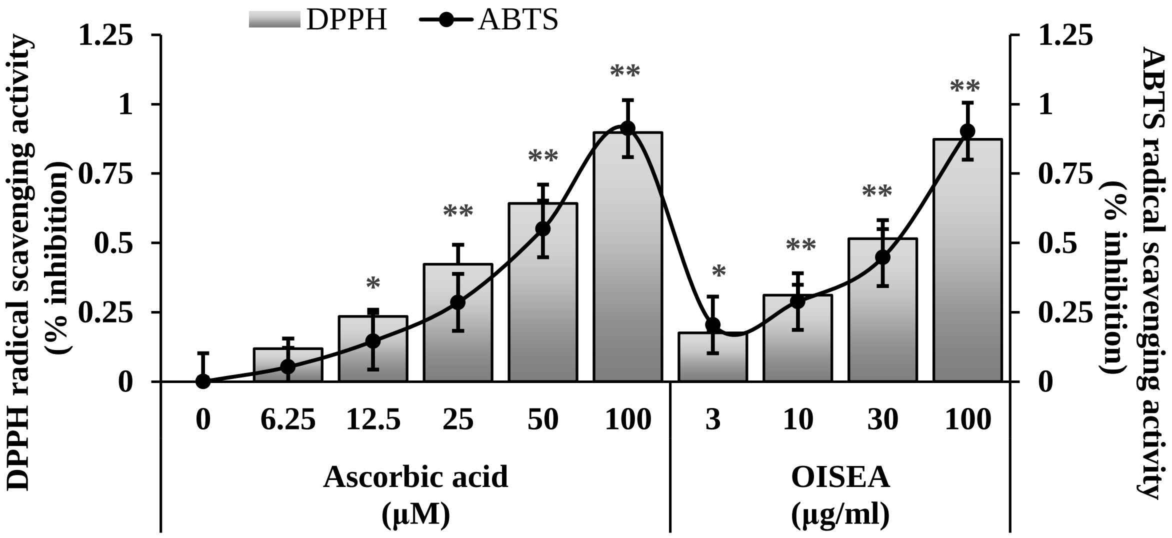

Figure S2: DPPH- and ABTS-radical scavenging activities of ethylacetate fractions of *Oroxylum indicum* seed . Values are expressed as the mean ± SD (n=3). *$p < 0.05$ and **$p < 0.01$, significantly different from control, using student's t-test.

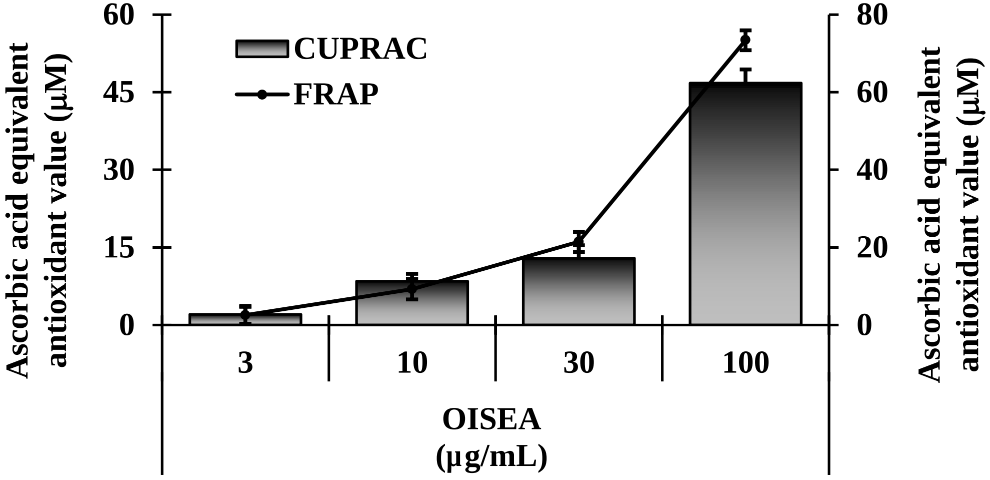

Figure S3: CUPRAC and FRAP activities of ethylacetate fractions of *Oroxylum indicum* seed . Values are expressed as the mean ± SD (n=3). *p < 0.05 and **p < 0.01, significantly different from control, using student's t-test.
